# Severity of Experimental Autoimmune Uveitis Is Reduced by Pretreatment with Live Probiotic *Escherichia coli* Nissle 1917

**DOI:** 10.3390/cells10010023

**Published:** 2020-12-25

**Authors:** Otakar Dusek, Alena Fajstova, Aneta Klimova, Petra Svozilkova, Tomas Hrncir, Miloslav Kverka, Stepan Coufal, Johan Slemin, Helena Tlaskalova-Hogenova, John V. Forrester, Jarmila Heissigerova

**Affiliations:** 1 Department of Ophthalmology, First Faculty of Medicine, Charles University and General University Hospital in Prague, 128 08 Prague, Czech Republic; ota.Dusek@seznam.cz (O.D.); aneta.klimova@volny.cz (A.K.); Petra.Svozilkova@lf1.cuni.cz (P.S.); jarmila.heissigerova@vfn.cz (J.H.); 2 Institute of Microbiology of the Czech Academy of Sciences, v.v.i., 142 20 Prague, Czech Republic; alena.fajstova@biomed.cas.cz (A.F.); coufal@biomed.cas.cz (S.C.); jojohas11@gmail.com (J.S.); tlaskalo@biomed.cas.cz (H.T.-H.); 3 Institute of Microbiology of the Czech Academy of Sciences, v.v.i., 549 22 Novy Hradek, Czech Republic; tomas@gnotolab.com; 4 Section of Immunology and Infection, Institute of Medical Sciences, University of Aberdeen, Aberdeen AB24 3FX, UK; j.forrester@abdn.ac.uk; 5 Immunology and Virology Program, Centre for Ophthalmology and Visual Science, The University of Western Australia, Crawley, Western Australia 6009, Australia; 6 Centre for Experimental Immunology, Lions Eye Institute, Nedlands, Western Australia 6009, Australia

**Keywords:** probiotics, *Escherichia coli* Nissle 1917, mucosal immune system, macrophages, experimental autoimmune uveitis

## Abstract

Non-infectious uveitis is considered an autoimmune disease responsible for a significant burden of blindness in developed countries and recent studies have linked its pathogenesis to dysregulation of the gut microbiota. We tested the immunomodulatory properties of two probiotics, *Escherichia coli* Nissle 1917 (EcN) and *E. coli* O83:K24:H31 (EcO), in a model of experimental autoimmune uveitis (EAU). To determine the importance of bacterial viability and treatment timing, mice were orally treated with live or autoclaved bacteria in both preventive and therapeutic schedules. Disease severity was assessed by ophthalmoscopy and histology, immune phenotypes in mesenteric and cervical lymph nodes were analyzed by flow cytometry and the gut immune environment was analyzed by RT-PCR and/or gut tissue culture. EcN, but not EcO, protected against EAU but only as a live organism and only when administered before or at the time of disease induction. Successful prevention of EAU was accompanied by a decrease in IRBP-specific T cell response in the lymph nodes draining the site of immunization as early as 7 days after the immunization and eye-draining cervical lymph nodes when the eye inflammation became apparent. Furthermore, EcN promoted an anti-inflammatory response in Peyer’s patches, increased gut antimicrobial peptide expression and decreased production of inducible nitric oxide synthase in macrophages. In summary, we show here that EcN controls inflammation in EAU and suggest that probiotics may have a role in regulating the gut–eye axis.

## 1. Introduction

Non-infectious (presumed autoimmune) uveitis is a sight-threatening inflammatory disease affecting people at any age, most commonly between 20 and 60 years of life [1,2]. Despite the introduction of many new treatment modalities, up to 10% of patients with uveitis become blind, making uveitis one of the leading causes of blindness in the developed world [1,3,4]. An association between inflammatory bowel disease (IBD) and some forms of uveitis has long been recognized while recent studies have linked the pathogenesis of non-infectious uveitis to dysregulation of the gut microbiome [5,6].

The gut microbiota is a broad ecosystem of bacteria, archaea, fungi, and viruses. Microbiota fundamentally influences the development and reactivity of the immune system and its perturbation, i.e., dysbiosis, is associated with several inflammatory and autoimmune disorders [7,8]. While some conditions, such as spondyloarthritis or inflammatory bowel diseases, are often associated with intraocular inflammation, several recent studies show that ocular inflammation is often accompanied by changes in the gut microbiome [9,10,11,12].

When considering the vast array of its biological activities and the availability of tools, the gut microbiota is a good target for therapy of disorders of the digestive tract. These tools include diet, nutrients, probiotics, prebiotics, antibiotics (ATB) and transfer of the whole microbiota community—fecal microbiota ‘transplantation’ [13,14]. ATB represent very powerful instruments for manipulation of the microbiota. Some of them can even induce long-lasting changes in microbiota, at least at low taxonomic levels [15]. Early life exposure to ATB not only alters the gut microbiota, but it also alters the host’s metabolism [16,17].

Recently, the gut microbiota was found to have protective influence on immune events in the eye [18]. The subsequent emergence of the “gut–eye” axis theory thus pointed to the gut microbiota as a possible modulator of ocular health. Indeed, alterations of intestinal microbiota change the systemic pro-inflammatory status of the host [8] as shown in animal models of ocular inflammation [19,20,21]. In our previous studies, we found that oral treatment of mice with a combination of antibiotics, including metronidazole, decreased the severity of retinal inflammation to a similar degree as that seen in germ-free mice [20]. Shifts in gut microbiota composition may influence the severity of inflammation by changing the concentration of short-chain fatty acids. These strong immuno-modulators produced by gut bacteria appear to prevent ocular inflammation by shifting the balance of T cells from pathogenic Th17 to tolerizing Treg and prevent the trafficking of lymphocytes between the gastrointestinal tract and the eye [22,23].

Probiotics are live microorganisms which confer a health benefit on the host, if administered in sufficient quantities [24]. The idea of using probiotics for health benefits was proposed in 1908 by Élie Metchnikoff [25] and successfully used in 1917 when *Escherichia coli* Nissle 1917 (EcN) was isolated and used to treat patients suffering from shigellosis [26]. In clinical practice, EcN proved to be effective in maintaining remission of ulcerative colitis, irritable bowel syndrome, acute diarrhea in infants and toddlers and uncomplicated diverticular disease of colon [27,28,29]. In experimental studies, its beneficial effect went beyond the gut-related pathologies and showed promising results in a murine model of multiple sclerosis (experimental autoimmune encephalomyelitis) and in the prevention of severe lung inflammation in a murine model of allergic asthma [30,31]. A protective effect on clinical and experimental extra-intestinal pathology, such as allergic asthma, is well described for another member of the same bacterial species *E. coli* O83:K24:H31 (EcO) [32,33,34].

Possible mechanisms of the beneficial effects of probiotics include: (1) antagonism through production of antimicrobial substances; (2) competition with pathogens for adhesion to the epithelium and for nutrients; (3) immunomodulation of the host; and (4) inhibition of bacterial toxin production [35].

In this study, we analyzed the anti-inflammatory potential of the above two probiotic *E. coli* strains in a murine model of experimental autoimmune uveitis (EAU). We find that oral EcN but not EcO reduces the severity of EAU when given as a preventive intervention and only as live bacteria. We demonstrate the importance of treatment timing and identify possible underlying immune mechanisms.

## 2. Materials and Methods

### 2.1. Animals

Female C57BL/6J mice (5 to 8 weeks old) were obtained from the breeding colony of The Center for Experimental Biomodels, First Faculty of Medicine, Charles University in Prague and housed at the conventional animal facility of the Department of Pharmacology, First Faculty of Medicine, Charles University in Prague. Mice with congenital defects, such as microphthalmia or cataract, were excluded from the experiments. The mice for each experiment arrived in bulk and were randomly distributed to the individual cages upon arrival. This study was carried out in accordance with the recommendations of the ethics standards defined by the EU legislation on the use of experimental animals (2010/63/EU) and the Czech animal welfare act. The protocols were approved by The Commission for Animal Welfare of the First Faculty of Medicine of Charles University in Prague, The Ministry of Education, Youth and Sports (MSMT 9993/2017-2).

### 2.2. Experimental Autoimmune Uveitis (EAU) Induction

EAU was induced by subcutaneous injection of interphotoreceptor retinoid-binding protein peptide 1–20 (IRBP; [Homo sapiens] H2N-GPTHLFQPSLVLDMAKVLLD-OH, New England Peptide, Gardner, MA, USA) 500 μg per mouse emulsified in CFA containing 3.3 mg/mL of heat-killed *Mycobacterium tuberculosis* H37Ra (Difco, Franklin Lakes, NJ, USA) immediately followed by intraperitoneal application of pertussis toxin (PTx; List Biological Laboratories, Inc., Campbell, CA, USA) 1.2 μg, as previously reported [36].

### 2.3. Probiotics

**Figure 1:**
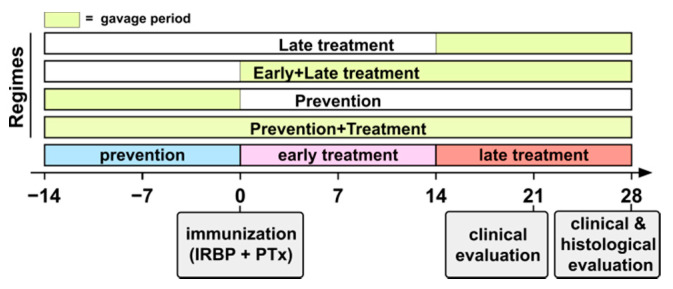
Experimental schedules.

Both probiotic bacteria (EcN, EcO) were cultured in Lennox’s version of Luria Bertani broth (Sigma-Aldrich, St. Louis, MO, USA). To obtain strain-specific growth curves, optical densities at 600 nm (A_600_) were measured by a spectrophotometer and compared by CFU counting after 24-h culture on 0.5% agar plates prepared from the same broth. Next, the fresh bacterial suspensions were diluted in sterile saline (0.9% NaCl; ARDEAPHARMA, a.s., Sevetin, Czech Republic) to a final concentration of 10^10^ mL^−1^ of either live *Escherichia coli* O83 (EcO; serotype O83:K24:H31; Dyntec spol. s.r.o., Terezin, Czech Republic) or live or autoclaved (121 °C, 30 min, 0.12 MPa) *Escherichia coli* Nissle 1917 (EcN; serotype O6:K5:H1; Ardeypharm GmbH, Herdecke, Germany). Each mouse received a dose of 100 μL (10^9^ bacteria) of this suspension. The viability of the bacteria was regularly monitored by culture on agar plates. Aliquots were stored at 4–8 °C for up to 2 weeks as a wet pellet (5 min, 2600× *g*, 4 °C) and resuspended with sterile saline immediately prior to administration. Treatments were applied 3 times per week using one of the four experimental schedules with a saline-treated control group as follows (Figure 1): (a) two weeks prior to EAU induction and continued for the duration of the experiment (combined prevention and treatment schedule, 18 doses); (b) two weeks prior to EAU induction-only (prevention schedule, 6 doses); (c) day 1 post EAU induction and continued for the duration of the experiment (early and late treatment schedule, 12 doses); (d) 14 days post EAU induction, late treatment-only schedule. In addition, (e) there was a control group of mice gavaged with 100 μL of saline solution (placebo group; no treatment schedule). To study the effect of EcN on the immune system before the EAU symptoms appear, we performed combined prevention and treatment schedule (a) but killed the mice 7 days after the EAU induction (9 doses).

### 2.4. Clinical Evaluation

A single image of the posterior central retinal part from each eye was taken during in vivo clinical examination (fundus biomicroscopy), which was performed on the 21st and 27th day after EAU induction using the TEFI imaging system [37]. An additional +4.0 diopter lens between the camera and the otoscope was used. During the procedure, the mice were under general anesthesia (ketamine 80 mg/kg and xylazine 5 mg/kg (both Bioveta, a.s., Ivanovice na Hane, Czech Republic)) administered intraperitoneally. The fundi were imaged through a dilated pupil (tropicamide, Unitropic 1% oph. gtt., Unimed Pharma, Bratislava, Slovakia) and phenylephrine (Neosynephrin-POS 10% oph. gtt., Ursapharm, Prague, Czech Republic). The otoscope was applied to the cornea using carbomer eye gel (Vidisic gel, Bausch and Lomb, Prague, Czech Republic) [38].

Retinal inflammatory changes were evaluated separately for the optic disc, retinal vessels, and retinal tissue changes in the posterior fundus of both eyes (central region) [39]. All images were evaluated independently by two experienced ophthalmologists (OD, AK), and where subjective differences occurred, a consensus grading of the two evaluations was used. The final clinical grade for each eye was then recorded (0 (no detectable changes) to 4 (most severe EAU)) according to the previously published criteria [20] and the score for each mouse is shown as the mean for both eyes.

### 2.5. Histological Evaluation

The mice were sacrificed on day 28 and the eyes were enucleated and immediately immersed in Tissue-Tek^®^ O.C.T. COMPOUND™ (Sakura Finetek USA, Inc., Torrance, CA, USA) and frozen in 2-methylbutane (Sigma-Aldrich, St. Louis, MO, USA) in liquid nitrogen. The samples were stored at −70 °C until sectioning to 7-μm-thick slices (at −17 to −21 °C). Sections were taken at 3 levels: both eye peripheries and centrally through the optic nerve. The samples were cut with a cryostat (Leica CM 1850) and stained with hematoxylin and eosin. The samples were then evaluated by two experienced ophthalmologists and graded using a standardized scoring system published previously [20]. The final histological grade for each mouse was obtained by averaging the grade for each eye ranging from 0 (no detectable changes) to 4 (most severe EAU).

### 2.6. Immunophenotyping by Flow Cytometry

Mouse mesenteric (mLN), inguinal (iLN) and cervical (cLN) lymph nodes were separately harvested after EAU induction and single-cell suspensions prepared as previously described [40]. To analyze gut macrophages, 2 cm of ileum without Peyer’s patches was harvested from mice 7 days after immunization and cells from lamina propria mucosae were isolated by mechanical disintegration and enzymatic digestion according to the previously published protocol [41,42]. Flow cytometric analysis was performed as follows: the cell suspensions were washed in phosphate-buffered saline (PBS), labeled with Fixable Viability Dye (ThermoFisher Scientific, Waltham, MA, USA), fixed and permeabilized with eBioscience™ Foxp3/Transcription Factor Staining Buffer Set (ThermoFisher Scientific), non-specific staining was blocked with 10% normal mouse serum and anti-CD16/CD32 antibody (Fc receptors) and stained either for T cells or monocytes and macrophages markers using fluorescence-labeled antibodies (Supplementary Table S1). To analyze intracellular cytokine production, cells (2 × 10^5^ cells in complete RPMI) were incubated either for 18 h with plate-bound 5 µg/mL anti-CD3ε and 4 µg/mL of anti-CD28 (both from Biolegend) or for 40 h with 20 µg/mL of IRBP and a mixture of 3 μg/mL of Brefeldin A and 2 μM Monensin (both ThermoFisher Scientific) was added for the last 4 h of incubation. Next, the cells were washed, labeled with Fixable Viability Dye (ThermoFisher Scientific), fixed and permeabilized with eBioscience™ Intracellular Fixation & Permeabilization Buffer Set (ThermoFisher Scientific), blocked with anti-CD16/CD32 antibody, and stained for CD3, CD4, CD8 (all Biolegend), IFN-γ, IL-17, and TNF-α (all ThermoFisher Scientific) (Supplementary Table S1). Flow cytometry data were obtained using an LSRII (BD Biosciences, San Jose, CA, USA) and analyzed using the FlowJo software (Tree Star Inc., Ashland, OR, USA). Gating strategies are shown on Supplementary Figure S1.

### 2.7. Analysis of Gut Cytokine Production

Next, Peyer’s patches and samples of colon tissue were cultured ex vivo, as described earlier [43]. Briefly, three distal Peyer’s patches and one three-millimeter punch biopsy from distal colons were collected, weighed and cultured in 500 µL of complete RPMI medium (Merck; Cat# R0883) containing 10% heat-inactivated fetal bovine serum (Biochrom GmbH, Berlin, Germany; Cat# S 0115) and 1% Antibiotic-Antimycotic solution (Merck) in a humidified incubator (37 °C, 5% CO_2_) for 48 h. The supernatants were collected and stored at −20 °C until analysis. Cytokines were measured in tissue culture supernatants using appropriate ELISA sets for murine TNF-α, IL-6, IL-1β, IL-33, and S100A8 (all Bio-Techne, Minneapolis, MN, USA; Cat# DY410, DY406, DY401, DY3626 and DY3059) according to the manufacturer’s instructions.

### 2.8. RT-PCR for Defensins and Other Regulatory Genes in the Gut

Colon and ileal biopsies were collected at the experiment termination and stored in RNA*Later* (Qiagen, Hilden, Germany) at −20 °C until analysis. Tissue was homogenized with ceramic beads Lysing Matrix D on FastPrep-24 (both MP Biomedicals, Solon, OH, USA) and total RNA was extracted with TRI reagent (Zymo Research, Irvine, CA, USA). RNA was then treated with a TURBO DNA-free Kit (ThermoFisher Scientific) to remove DNA and the concentration and quality was measured with a NanoDrop 2000 (ThermoFisher Scientific). All samples were then diluted to equal concentrations of RNA and 500 ng of RNA were transcribed to cDNA using a SuperScript IV Reverse Transcriptase kit (ThermoFisher Scientific), Oligo(dT)12-18 Primer (Generi Biotech s.r.o., Hradec Kralove, Czech Republic) and RNaseOUT™ Recombinant Ribonuclease Inhibitor (ThermoFisher Scientific). Quantitative PCR was performed with gp SG PCR Master Mix (Generi Biotech, Hradec Kralove, Czech Republic) and specific primers (Supplementary Table S2) on a LightCycler^®^ 480 Real-Time PCR System [44] to determine changes in mRNA expression. The cycling parameters were as follows: 3 min at 95 °C, 39 cycles of 30 s at 94 °C, 40 s at 60 °C and 1 min at 72 °C. Data were normalized to the reference gene eukaryotic elongation factor 2 (*Eef2*) and relevant control group and changes in gene expression were calculated according to the 2^−ΔΔCt^ method.

### 2.9. Analysis of Probiotic Colonization Ability

To assess the ability of EcN and EcO to colonize murine intestine, we administered EcN or EcO by gavage to C57BL/6J mice (single dose of 10^9^ live bacteria per mouse in 100 µL of saline; EcN *n* = 5; EcO *n* = 4). To prevent cross-contamination, EcN- and EcO-treated mice were housed separately. After 48 h, mice were killed, and the intestine content was collected from the ileum, caecum and colon. Each sample was weighed, DNA isolated using MasterPure DNA Purification Kit (Epicentre, Madison, WI, USA) and reconstituted to 5 ng/μL. Serial dilutions of DNA from 5 × 10^8^ CFU of the original suspensions were used as standards. Next, 4 µL of this template were mixed with gb SG PCR Master Mix (Generi Biotech s.r.o.) and specific primers (400 nM, Supplementary Table S2). The cycling parameters were as follows: 4 min at 95 °C, 40 cycles of 30 s at 94 °C, 30 s at 61 °C and 60 s at 72 °C. Probiotic counts were adjusted to CFU per gram of sample and statistically analyzed. In these experiments, we found that both EcO and EcN are present in the ileum, cecum and colon of colonized mice in similar quantities out to 48 h after gavage (Supplementary Figure S2). Thus, feeding mice with live probiotics every 2 days should allow continuous exposure. However, neither EcO nor EcN were detectable in the gut 5 days after the gavage, showing that they do not colonize the small or large intestine for a prolonged time.

### 2.10. Generation and Culture of Bone Marrow-Derived Macrophages (BMDM)

Bone marrow was harvested from the femurs of 10-week-old C57BL/6J mice and single cell suspensions prepared as described previously [45]. The cells were then grown for 6 days in bone marrow differentiation media (DM) with medium exchange d4 of culture. DM comprises complete RPMI medium (Merck) containing 10% heat-inactivated fetal bovine serum (Biochrom GmbH), 1% Antibiotic-Antimycotic solution (Merck), 50 µM 2-mercaptoethanol (Merck) and was supplemented with L929 cell (ATCC CCL-1) conditioned media. L929 cell conditioned medium contains colony-stimulating factor 1 (CSF-1) and was produced by growing L929 cells in complete RPMI medium for 10 days. On d6 of culture, adherent cells were harvested and re-plated in 96F-well plates at 1 × 10^6^ cells/mL and incubated overnight to allow cells time to adhere. The following day, macrophages were stimulated with 1, 10, 100, or 1000 µg/mL of either EcN or EcO lysate in a humidified incubator (37 °C, 5% CO_2_) for 24 h. Either lysate was prepared by passing the suspensions 3 times through a French Press, lyophilized, re-suspended in complete RPMI medium, and tested for sterility.

### 2.11. Data Analysis

Clinical and histological EAU scores from both eyes of the same mouse were averaged and data were analyzed using GraphPad Prism (ver. 8.1.1.; GraphPad Software, San Diego, CA, USA). A nonparametric Mann Whitney test was used to evaluate the differences between placebo- and probiotic-treated groups, 2-way ANOVA with Bonferroni’s multiple comparisons test was used to test the differences between EcO and EcN lysates on BMDM and a *p*-value of *p* < 0.05 was considered significant. The data are presented as individual data points with the arithmetic mean in data showing clinical and histological score, as geometric mean (bar) and geometric standard deviation (error bar) in data showing expression and as arithmetic mean (bar), or as standard deviation (error bar) for all other graphs.

## 3. Results

### 3.1. Colonization with EcN But Not EcO Is Protective against Development of EAU

**Figure 2:**
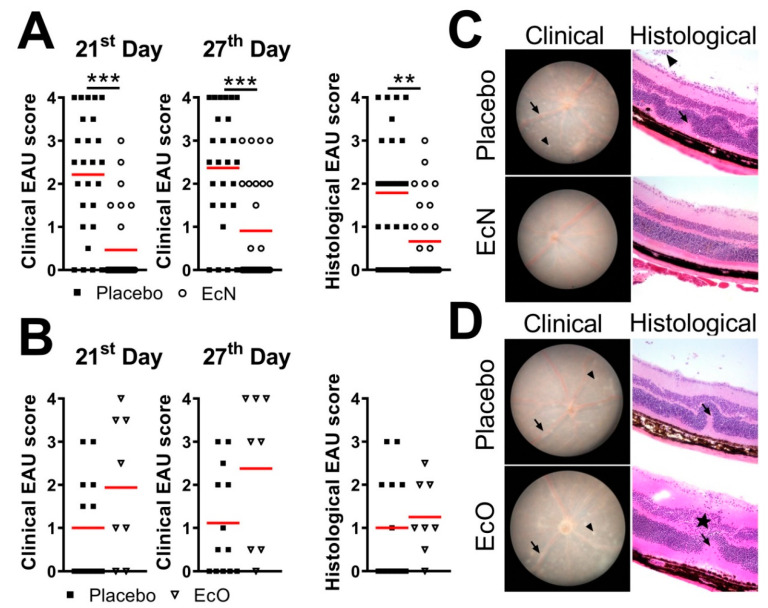
Live probiotic EcN protects from severe forms of EAU when administered starting 14 days before the immunization until day 28 post-induction [*n* = 26 (placebo), 28 (EcN) in total] (**A**), while EcO has no effect [*n* = 13 (placebo), 8 (EcO) in total] (**B**). EAU severity was assessed by in vivo fundus biomicroscopy on the 21st and 27th day post-induction and by histological analysis 28th day post-induction and the differences were quantified by unpaired Mann Whitney test; graphs show individual values and red lines represent mean, ** *p* < 0.01 *** *p* < 0.001. Data are pools from 5 (EcN) or 2 (EcO) independent experiments. Representative pictures from clinical and histological pictures from the 27th day show typical pathological features when mice were treated with EcN (**C**) or EcO (**D**). These include chorioretinal lesions (arrowhead), vascular sheathing (arrow) and optic nerve inflammation during clinical examination and infiltration of cells in the inner retina (star), cell infiltration of the vitreous body (vitritis, arrowhead) and retinal folds (arrow) during histological examination (optical magnification 200×).

When EcN was administered to mice in the preventive regime (starting 14 days before the EAU induction and continuing for 28 days post immunization with IRBP), it significantly decreased the severity of EAU both clinically and histologically (Figure 2A,C). In contrast, similar colonization with EcO did not protect mice from EAU and even showed a slight tendency to worsen its severity (Figure 2B,D) with significant mortality (55.6%).

### 3.2. Autoclaved EcN Loses Its Ability to Mitigate EAU

**Figure 3:**
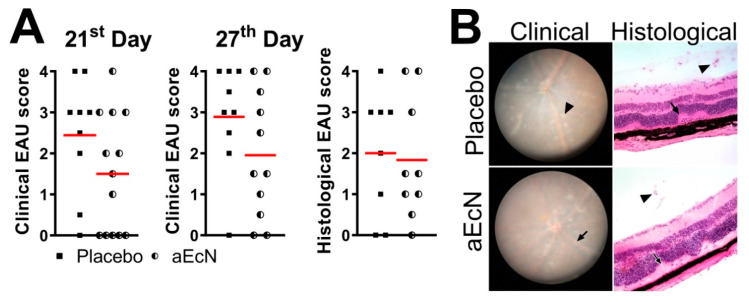
Treatment with autoclaved EcN from 14 days before EAU induction until day 28 post-induction is less effective than with live bacteria, graphs show individual values and red lines represent mean. (**A**) Data are pools from 2 independent experiments (*n* = 9 (placebo) or 13 (aEcN) in total) and differences were quantified by unpaired Mann Whitney test. (**B**) Representative images of clinical and histological findings with typical findings marked, i.e., clinically as chorioretinal lesions (arrowhead) and vessel sheathing (arrow) and histologically as inflammatory cell infiltration of the vitreous body (vitritis, arrowheads) and retinal folds (arrows) histological (optical magnification 200×).

Next, we assessed whether viable EcN was required for a beneficial effect on EAU severity. Treatment of mice with autoclaved EcN (aEcN) had a small but non-significant effect on EAU severity, suggesting that viable organisms are required to protect against EAU (Figure 3). This suggests that an EAU suppressive effect is dependent on live probiotic bacteria.

### 3.3. Treatment with Live EcN Is Only Effective When Given Prophylactically, i.e., Prior to Induction of EAU

**Figure 4:**
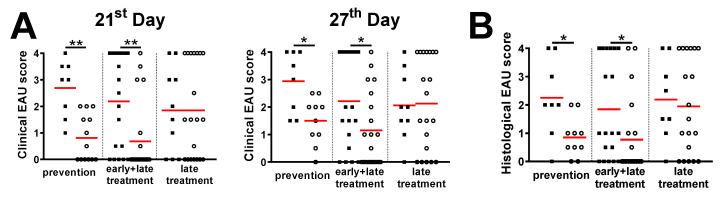
Live EcN decreases severity of EAU if administered at time of disease induction. The EAU severity was analyzed by in vivo fundus biomicroscopy on the 21st and 27th day post-induction (**A**) and by histological analysis at day 28 post-induction (**B**) and the differences were quantified by unpaired Mann–Whitney test; * *p* < 0.05 ** *p* < 0.01; *n* = 8 (placebo prevention), 13 (EcN prevention), 19 (placebo early + late treatment), 22 (EcN early + late treatment), 10 (placebo late treatment) and 20 (EcN late treatment) in total, graphs show individual values and red lines represent mean. Black squares represent individual inflammation severity scores from the placebo group and open circles from the EcN group.

To determine whether treatment with EcN is effective in the active or prodromal stages of EAU, we administered live EcN using different experimental schedules. We found that live EcN is effective in mitigating EAU severity only if administered before or from the time of EAU induction (Figure 2 and Figure 4). Interestingly, exposure to EcN before EAU induction is equally effective irrespective of whether treatment is only prophylactic (prior to immunization) or is continued after EAU induction (Figure 2 and Figure 4) but is not effective when started 14 days after immunization (late treatment). This suggests that EcN prevents the induction EAU during the time of IRBP antigen presentation but has little effect once T cell activation has been established. Similarly, as for the preventive + treatment regime, autoclaved EcN were ineffective whether administered 14 days before immunization or from the time of immunization (data not shown).

### 3.4. Probiotic Treatment with EcN Decreases the IRBP-Specific Response of Helper T Cells in the Inguinal, Mesenteric and Cervical Lymph Nodes But Has No Effect on Regulatory T Cell Numbers or General T Cell Stimulability

Next, considering their role in autoimmune inflammation, we measured proportions of Th17, innate lymphoid cells (ILC) 3 and regulatory T cells (Tregs) in mesenteric (mLN) and cervical (cLN) lymph nodes. Oral EcN did not influence the proportion of these cells (Supplementary Figure S3), suggesting that they do not play any significant role in the ability of EcN to mitigate the EAU.

Moreover, when stimulated with TCR stimulant (anti-CD3/anti-CD28), cells from EcN-treated mice exhibited a similar potential to produce typical pro-inflammatory cytokines as cells from placebo-treated mice. Only a small increase in the proportion of TNF-α^+^-expressing cytotoxic T cells in mLN of EcN-treated mice was observed (Supplementary Figure S4A) but was not supported by increased levels of cytokine production (Supplementary Figure S4B). This finding is therefore of low significance.

**Figure 5:**
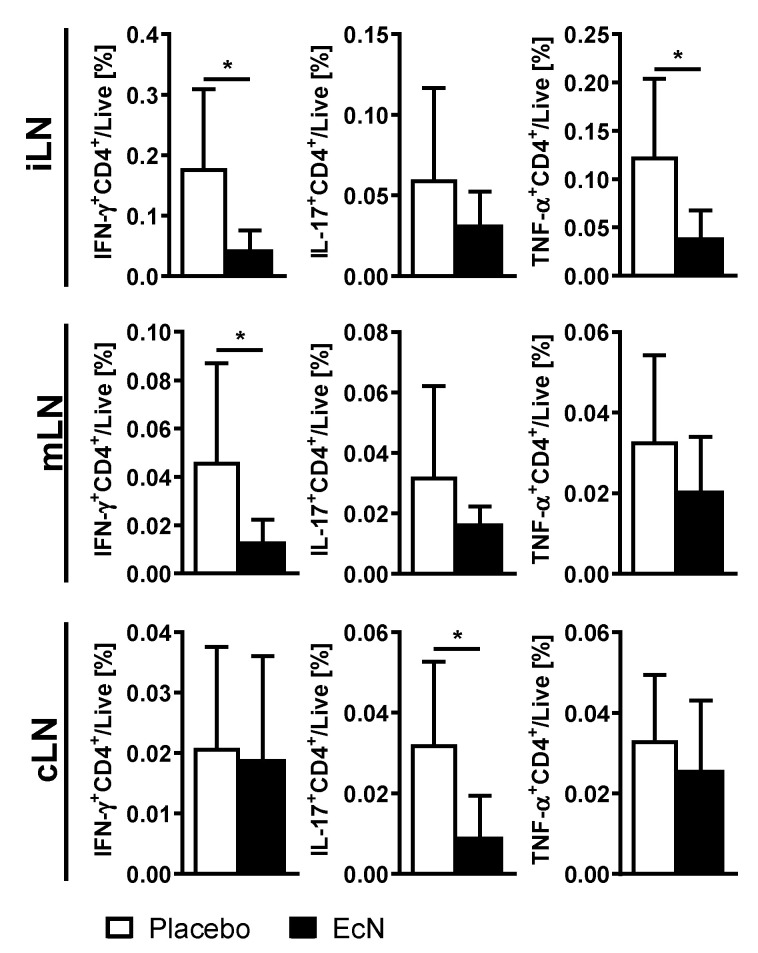
Oral live EcN suppresses the response of helper T cells to IRBP in the inguinal, mesenteric and cervical lymph nodes at day 28 post-induction, as measured by FACS. Differences were quantified by unpaired Mann Whitney test; * *p* < 0.05 (*n* = 5–8 per group).

Next, we asked whether EcN changes the response to IRBP in cells isolated from inguinal, mesenteric and cervical lymph nodes 28 days after EAU was induced with IRBP. We found that, in general, T cells from mice treated with EcN respond less to IRBP stimulation in vitro. These differences were statistically significant in iLN for IFN-γ and TNF-α, in mLN for IFN-γ and in cLN for IL-17 (Figure 5). These data suggest that although there is no difference in general Th1/Th17 response, oral EcN renders T cells less responsive in vitro specifically to IRBP after challenge in vivo, suggesting an effect on antigen presentation and priming of T cells.

### 3.5. EcN Probiotic Promotes an Anti-Inflammatory Response in Peyer’s Patches and Increases Antimicrobial Peptide Expression

**Figure 6:**
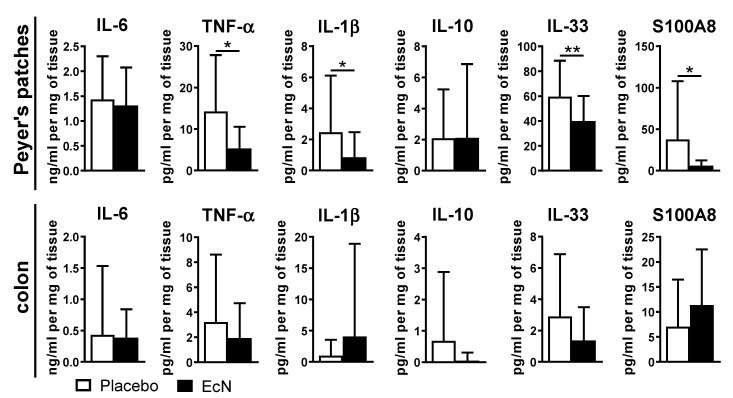
Analysis of proteins in supernatants of cultured cells from Peyer’s patches and colon tissue at day 28 post-induction. Live oral EcN decreased the production of several pro-inflammatory cytokines in Peyer’s patches but not in the colon. Data are pooled from 5 independent experiments (*n* = 25 per group in total) and differences were quantified by unpaired Mann–Whitney test; * *p* < 0.05 ** *p* < 0.01.

**Figure 7:**
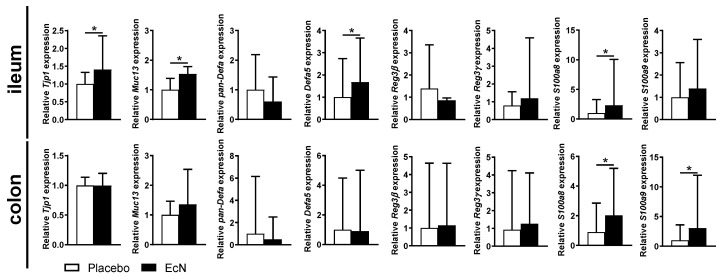
Live EcN increases the expression of some anti-microbial peptides in the gut at day 28 post-induction. Data are pooled from 5 independent experiments (in total: *n* = 25 (placebo) or 27 (EcN)) and differences were quantified by unpaired Mann–Whitney test; * *p* < 0.05.

We next assayed cytokine production in EcN-treated mice (vs. placebo) in the supernatant of cultured Peyer’s patches or colon tissues by ELISA (see methods). While oral live EcN did not change the production of pro-inflammatory factors in the colon, decreased production of TNF-α, IL-1β, IL-33, and S100A8 in Peyer’s patches of EcN-treated mice was observed (Figure 6). However, when we assayed gene expression in ileal tissues (without Peyer’s patches) and the colon, we found similar levels of expression of *S100a8* in both the ileum and colon although *S100a9* was increased only in the colon (Figure 7). 

We also assayed for anti-microbial peptide genes in both tissues. EcN increased expression of *Defa5* (cryptidyn 5), *Muc13* and *Tjp1* in the ileum but not the colon but did not change the expression of α defensins in general or other anti-microbial peptides, such as *Reg3β* or *Reg3γ*, in either compartment (Figure 7). The changes in *Defa5* (cryptidyn 5), *Muc13* and *Tjp1* may influence gut barrier integrity while providing an anti-inflammatory defense in Peyer’s patches. While there was a small increase in *Tlr5* expression in the colon, there were no differences in *Irak3* or *Defb4* expression in either tissue (Supplementary Figure S5).

### 3.6. Live EcN Decreases the Proportion of Classically Activated Macrophages

**Figure 8:**
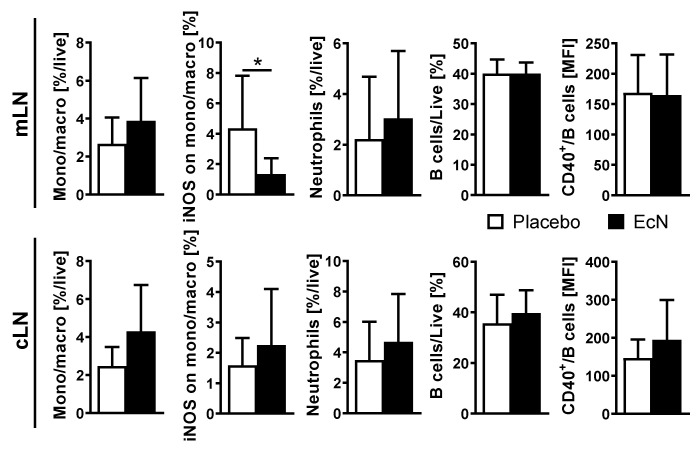
Flow cytometric analysis of myeloid cells in gut-draining and eye-draining lymph nodes (mLN and cLN, respectively) at day 28 post-induction. Live EcN treatment decreased the proportion of iNOS+ monocytes/macrophages in mLN but not in cLN. Data are pooled from 2 independent experiments (*n* = 11 per group in total) and differences were quantified by unpaired Mann Whitney test; * *p* < 0.05. The monocytes/macrophages are defined as live cells CD45^+^CD11c^−^B220^−^CD3^−^CD49b^−^Ly^−^6G^−^SSC^lo^, neutrophils are defined as live cells CD45^+^CD11c^−^B220^−^CD3^−^CD49b^−^Ly^−^6G^+^ and B cells are described as live cells CD45^+^CD11c^−^B220^+^, as described earlier [47].

To assess myeloid and B cell status after EcN treatment, mice treated by the preventive plus treatment schedule (see Figure 1) were assessed on day 28. There were no differences between the groups in the proportion of monocytes/macrophages overall in the mLN between EcN-treated and placebo-treated mice by flow cytometry in the mLN of EcN-treated mice than control mice. However, we observed a significantly lower proportion of inducible nitric oxide synthase (iNOS)^+^ macrophages (putative M1 macrophages) in the EcN-treated mice. Interestingly, these differences were not observed in the cLN draining the eye (Figure 8). Furthermore, there were no differences between the groups in the proportion of neutrophils or B cells in either lymph node set. The interactions between CD40 on the B cell and the CD154 on the T cell have multiple consequences for both types of cells [46]. However, there were no differences in CD40 expression between B cells from EcN and placebo-treated mice in either type of lymph node, suggesting that there is no involvement of B cells in the EcN effect on EAU.

### 3.7. EcN-Induced Immunoregulation Precedes the Development of EAU

**Figure 9:**
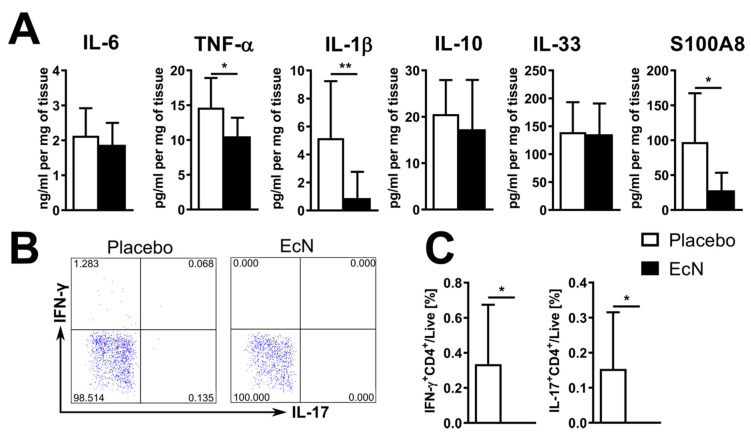
Live oral EcN decreases the production of several pro-inflammatory factors in non-stimulated Peyer’s patches at day 7 post-induction, as measured by ELISA (*n* = 9 (placebo) or 8 (EcN)) (**A**). At the same time, live EcN suppressed IFN-γ and IL-17 production by the IRBP-stimulated helper T cells in the inguinal lymph nodes, as shown by the typical dot plot gated on live CD3^+^CD4^+^ cells (**B**) and statistics from 8 (placebo) or 3 (EcN) mice as measured by flow cytometry (**C**). Differences were quantified by unpaired Mann–Whitney test; * *p* < 0.05, ** *p* < 0.01.

To assess the immune response in the gut during the prodromal stages of EAU development, we analyzed the effect of EcN on the gut immune system 7 days after induction. Moreover, we analyzed the IRBP-specific T cell response in the iLN to examine how oral EcN effects on the gut might influence the immune system at distance, in particular at lymph nodes draining the site of immunization. At this time (7d), there were no pathological changes in the eye. However, we found that treatment with live EcN induced anti-inflammatory changes in the gut on day 7 similar to those on day 28. EcN significantly decreased production of TNF-α, IL-1β and S100A8 in cells cultured from Peyer’s patches of EcN-treated mice at 7d (Figure 9A) without significantly changing their production in the colon or ileum (data not shown). When compared to controls, EcN-treated mice had a significantly lower proportion of macrophages in the ileum and in the mLN (Supplementary Figure S6). We also evaluated the status of the macrophage populations on the basis of pro-inflammatory (putative M1) vs. regulatory (putative M2) macrophages. There were no M2-type macrophages (CD38^+^Egr2^+^F4/80^+^CD45^+^) in the ileum of either group or in mLN of EcN-treated mice. At the same time, Th cells from lymph nodes draining the site of immunization [40] from EcN-treated mice failed to produce IFN-γ or IL-17 in response to IRBP stimulation in vitro (Figure 9B,C). There were no differences between IRBP-stimulated cells from mLN or cLN (data not shown). Even when stimulated in vitro, EcN lysate induces significantly weaker production of IL-1β than EcO lysate (Supplementary Figure S7). These data suggest that the anti-inflammatory effect of oral EcN commences during the prodromal stages of EAU development, at a time when active antigen presentation and effector T cell activation is occurring in the draining lymph node of the site of immunization prior to effector T cell egress and entry to the target organ by crossing the blood-retinal barrier of the eye. These systemic effects appear to be secondary to the anti-inflammatory effects of EcN predominantly in the Peyer’s patches.

## 4. Discussion

Gut microbiota drive the development of both protective and regulatory immune mechanisms [7] that influence immune reactivity systemically [20,48,49]. Multiple candidates with immuno-regulatory functions have been identified among the different gut phyla and species. In our previous study, we showed that the absence of microbiota or decreased bacterial load before induction of inflammation significantly decreases the susceptibility to EAU [20,38]. Here, we focused on the clinically more relevant approach of treating EAU with probiotic bacteria, which have established anti-inflammatory potential.

Both EcN and EcO have previously been shown in other models to have anti-inflammatory properties [30,31,34,50]. We found that while treatment with live EcN protects against EAU, treatment with live EcO does not. In fact, we found significant mortality among the EcO-treated animals. EcO has proven to be safe both in multiple animal models [34,50] and in humans [32,51], but it has the ability to produce multiple virulence factors [52]. This is not uncommon among otherwise safe microorganisms and these factors often improve their “fitness” and opportunities to colonize the gut. This effect is not commonly reported for EcO and may be caused by virulence factors released due to unique interactions between EcO and gut microbiota in any particular animal facility [53,54]. It does not seem that the EcO-induced mortality is related to use of strong adjuvants (CFA and PTx) as some mice in our experiments succumbed even before the immunization. There is variability in EAU severity between the two independent experiments we performed with EcO, but both show similar trends. It is well recognized that animal models of autoimmune diseases show considerable variability and EAU is no exception from this rule, as corroborated by many others [55]. In our work, we have shown statistical validity, using 8 or more mice per group in each experiment. Therefore, while the data do not show statistically that EcO has a deleterious effect on EAU, EcO clearly does not have a beneficial effect on EAU unlike EcN, which does (Figure 1). The differences in biological activity between these two closely related microbes further stress the fact that each isolate may trigger different mechanisms, thus leading to different outcomes, suggesting that the effectivity of probiotics may be disease specific and dependent on the particular pathogenic mechanisms [56].

The timing of the probiotic intervention may also influence pathogenic mechanisms. We compared four regimens of EcN administration. In one regimen, EcN was administered for the entire duration of the experiment (from 14 days before EAU induction to 28 days post induction). The remaining three regimens involved administration for set time periods: 14 days before EAU induction only (prevention); from the time of induction to the end of the experiment (28 days, early and late treatment); and for the last 14 days of the experiment (late treatment). We found that oral EcN decreases the severity of EAU only if administered before and during induction, suggesting that EcN influences initial antigen presentation and T cell priming. It is possible that the lack of an effect of late EcN treatment (i.e., starting at day 14) is due to the lower cumulative dose of EcN. However, this is unlikely since a similar 14-day treatment before EAU induction was sufficient to prevent severe EAU. Moreover, EcN does not colonize the mouse gut for more than a few days. Our finding that exposure to EcN only during 14 days before the induction is effective in EAU prevention is in agreement with a recent study finding that colonization with EcN could exert its beneficial effect beyond a local effect on the gut and prevent severe forms of experimental autoimmune encephalomyelitis [57].

Although we found that non-living microbial components can prevent severe intestinal inflammation [43,58], in the current experiments, only live bacteria were truly effective in suppressing induction of EAU. This suggests that either EcN prevents EAU by heat-sensitive factors or that prolonged interaction or production is necessary for the full effect. It may also be that a direct local beneficial effect of EcN is possible in intestinal inflammation, whereas for a distant/systemic effect, live bacteria and bacterially synthesized products, such as short-chain fatty acids, or other micronutrients, such as folate, are required, both of which require active colonization.

There are several mechanisms whereby probiotics can exert their anti-inflammatory potential. First, they could compete, communicate or target other microbes and some of them may have a strong influence on the host’s immune system [59,60]. For example, gut microbes prevent severe colitis by enhancing the integrity of the gut mucosal epithelium or may attenuate uveitis by modulating T cell phenotype and trafficking through the action of short-chain fatty acids [22,61,62]. Alternatively, this effect could be indirect via the host, by stimulating the immune system to produce anti-microbial peptides [59]. For these effects, long-term colonization may not be strictly necessary [34,63]. In fact, certain antigens from intestinal commensal bacteria are proposed to activate retina-specific autoreactive T cells [19], suggesting that some gut bacteria may have a negative effect and be among uveitis triggers. Second, they may improve gut barrier function, thus preventing the uncontrolled transfer of microbial antigens across mucosae (i.e., “leaky gut”) and pro-inflammatory tuning of the immune system [31,64]. Third, they may interact with the mucosal immune system, thus spreading the process of anti-inflammatory tuning further to cells of the innate and adaptive immunity [65,66].

Mechanisms whereby beneficial microbes could influence autoimmune processes include induction of regulatory T cells or attenuation of pro-inflammatory Th17 cells [43,67,68,69]. Decreased severity of EAU is accompanied by an increased proportion of FoxP3^+^CD4^+^ T cells and a decreased proportion of IL-17A^+^CD4^+^ T cells in cLN of germ-free mice [20]. Here, we did not find increased IL-17 production or raised percentage of IL-17-producing cells in the mLN or cLN accompanying the therapeutic effect of EcN in EAU. However, we found that EcN decreases the proportion of IRBP-specific IFN-γ^+^CD4^+^ and IL-17A^+^CD4^+^ T cells in inguinal lymph nodes, draining the site of immunization, as early as 7 days after the immunization, but does not change the numbers of these cells in mLN and cLN after IRBP stimulation at this time. However, by day 28, we found fewer IRBP-specific Th17 cells infiltrating the eye-draining cLN of EcN-treated mice. Different T cell-dependent mechanisms may be employed by other probiotics, as oral treatment with a mixture of probiotic bacteria, including *Lactobacillus casei*, *L. acidophilus*, *Lactobacillus reuteri*, *Bifidobacterium bifidum* and *Streptococcus thermophilus*, not only improves EAU, but it also increases the number of regulatory T cells and decreases IL-17^+^ and IFN-γ^+^ cytotoxic T cells in cLN [21]. This may be due to the fact that butyrate isa strong inducer of regulatory T cells, and wild-type EcN, unlike many beneficial microbes, does not produce detectable butyrate levels [70].

We also explored the immune microenvironment of the gut–eye axis. We found that EcN significantly decreases the production of multiple pro-inflammatory factors (TNF-α, IL-1β, IL-33 and S100A8) in Peyer’s patches. Interestingly, this anti-inflammatory effect was strictly localized to this tolerogenic inductive site of the mucosal immune system while similar effects were not apparent in the effector sites, such as the colonic mucosa. This compartmentalization of the mucosal immunity is important for simultaneous immune system regulation and immune protection [71]. A decrease in IL-1β is of particular interest, as it critically influences antigen priming during early EAU pathogenesis [72]. This is in agreement with our data on the timing of the probiotic intervention, as EcN seems to be effective only during the early stages of EAU induction. S100A8 is a myeloid cell-derived alarmin released as a damage-associated molecular pattern (DAMP), which stimulates leukocyte recruitment and maturation [73]. Its decreased production in the Peyer’s patches explains decreased macrophage activation, decreased inflammasome pathway activity and lower secretion of IL-1β, which result in reduced levels of antigen presentation, decreased IRBP-specific response and a milder form of EAU. This may be relevant even to human disease, as circulating S100A8/A9 were recently proposed as a biomarker of intraocular inflammation in uveitis patients [74]. We may even speculate that decreased S100A8 production in Peyer’s patches may be the cause for the reduction in other pro-inflammatory cytokines. Locally produced S100A8/A9 is an endogenous TLR4 agonist that amplifies the inflammatory reaction, thus keeping TNF-α and IL-1β high [75,76].

Gut dysbiosis, shifts in ileal defensin production and increased gut permeability are all features of EAU [77]. Interestingly, EcN not only left the gut epithelium intact, but it also induced protective mechanisms within the gut mucosa that prevented damage induced by enterohemorrhagic or uropathogenic *E. coli* [64]. EcN can improve gut barrier function by inducing colonic production of the antimicrobial peptide, human beta-defensin 2, in a flagellin-TLR5-dependent manner via NF-κB and AP-1 signaling pathways [78,79]. Interestingly, flagellin also induces *IRAK3* expression in cultured intestinal epithelial cells, which is associated with tolerance induction [80]. Microbiota-induced strengthening of the gut barrier is a mechanism that can influence different pathological processes in the gut and liver and may even be employed in fundamental processes, such as age-related “leaky gut” [81,82,83]. Therefore, we analyzed ileal and colonic expression of the flagellin sensor *Tlr5*, the negative regulator of TLR signaling *Irak3*, the gene for tight junction protein ZO-1 (*Tjp1*), *Muc13,* and a set of anti-microbial peptides, including the murine functional orthologue of human beta-defensin 2, *Defb4* [84]. While we were not able to detect *Defb4* in either the ileum or colon, we found increased expression of *Tlr5* and *S100a8* in the colon and *S100a8*, *Defa5* and *Muc13* in the ileum of the EcN-treated mice. Furthermore, we did not find changes in other anti-microbial peptides, such as defensins a, *Reg3β* and *Reg3γ*, suggesting that changes in the anti-microbial peptide production are selectively specific. Together with slightly increased ileal *Muc13* and *Tjp1* expression, these changes may suggest increased mucosal protection, which does not involve negative regulation by *Irak3*.

Of particular note is the difference in decreased S100A8 production in Peyer’s patches and its increased expression in the rest of the mucosa. Again, this suggests the importance of the compartmentalization of the mucosal immune response, which could regulate the response to antigen without compromising mucosal protection. In fact, others have reported that ileal *S100a8* expression is increased during EAU [77], suggesting that increased *S100a8* expression protects the gut barrier from failure and that EcN improves this protection by increasing the production of anti-microbial compounds.

The protective effect of EcN on the gut barrier is well established in multiple animal models [30,62,77,85]. We may speculate that other barriers, such as the blood-retinal barrier, are also strengthened after the oral administration of EcN, with tight junctions being a fundamental component of both [86]. While the microbial factors responsible for these effects are still unknown, they may involve outer membrane vesicles produced by EcN that can reinforce the epithelial barrier, possibly even on distant epithelium [87]. This is in agreement with data from a protease-induced murine model of asthma, where EcN prevented failure of the lung mucosal barrier [31]. The efficiency of EcN in both autoimmune and allergic inflammation may support its independence of T cell function, which we observed in our experiments. Moreover, microglia maintain blood-retinal barrier function during the initiation of ocular inflammation, thus preventing autoreactive lymphocytes from damaging the retina [88]. Since local barrier breakdown is a hallmark of inflammation, the suppression of inflammation in EAU by EcN treatment indicates that breakdown of the blood retinal barrier is reduced by this treatment. It is as yet unresolved whether EcN influences the blood-retinal barrier directly or indirectly in this process. Here, we might speculate that it is the latter, since EcN is only effective if administered around the time of immunization.

Interestingly, the beneficial effect of EcN on inflammation in EAU is mirrored in its effects on macrophages within the gut lymphoid system. While the mLN of both placebo- and EcN-treated mice contained similar proportions of macrophages, live EcN decreased their production of iNOS, thus reducing the proportion of pro-inflammatory M1 macrophages. This complements other data in this paper showing a reduced production of IL-1β and TNF-α in EcN-treated mice, as both these cytokines are associated with polarization of macrophages to an M1 phenotype [89].

## 5. Conclusions

In this study, we showed that live oral probiotic EcN, but not EcO, protects mice from EAU. This effect is seen only with live bacteria and only if administered before or around the time of EAU induction. Interestingly, this protective effect is accompanied by strengthening of the gut mucosal integrity, with a turn towards anti-inflammatory tuning of the gut mucosal immune system and a shift in the balance from the M1 to M2 type of macrophages. These changes appear to affect antigen presentation and priming of IRBP-specific T cells after challenge in the lymph nodes draining the site of immunization, and subsequently the severity of EAU. These results demonstrate a set of specific mechanisms affecting the gut–eye axis in controlling inflammation and suggest potential immuno-modulatory strategies for the prevention of ocular inflammation.

## Data Availability

The data presented in this study are available in the Supplementary Dataset.

## Supplementary Materials

The following are available online at https://www.mdpi.com/2073-4409/10/1/23/s1, Figure S1: Gating strategy, Figure S2: Probiotic EcO and EcN colonize mice used in our experiments for at least 48 h after a single dose, Figure S3: Live probiotic EcN does not change proportion of regulatory T cells, Th17 cells or innate lymphoid cells in mLN or cLN at day 28 post-induction, Figure S4: Oral EcN has no effect on expression of pro-inflammatory intracellular cytokine of anti-CD3/CD28 activated helper or cytotoxic T cells in mLN or cLN, Figure S5: Live EcN increases the expression of *Tlr5* in the colon at day 28 post-induction, Figure S6: Live EcN decreases proportion of macrophages and shift their M1 at day 7 post-induction, Figure S7: EcO lysate induces higher IL-1β production in BMDM compared to EcN lysate. Table S1: List of antibodies, Table S2: Primers used in qPCR and RT-PCR. Supplementary Dataset.
