# Supplementary material for: Severity of Experimental Autoimmune Uveitis Is Reduced by Pretreatment with Live Probiotic Escherichia coli Nissle 1917
Source: Cells. 2020 Dec 25;10(1):23. doi: 10.3390/cells10010023 (PMC7823395; doi:10.3390/cells10010023)
Supplement: Supplementary file 1 [file cells-10-00023-s001.zip › cells-916613-supplementary figure.pdf]

cells
MDPI

Supplementary Materials:

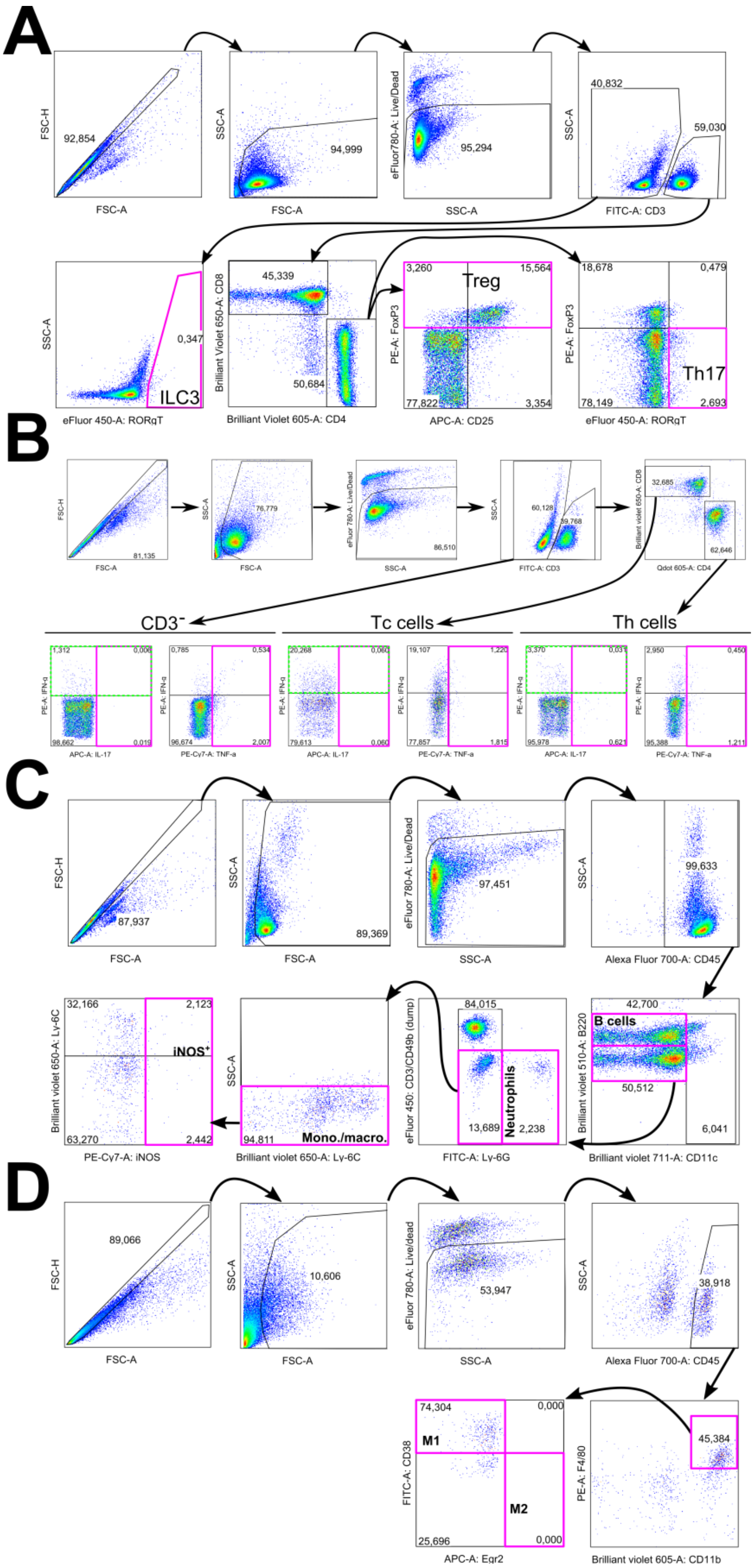

Figure 1. Gating strategy for regulatory T cells, Th17 and ILC3 (A), cytokine production by T cells (B) and monocytes, macrophages and neutrophils (C) using mesenteric lymph nodes (mLN) of placebo-treated C57BL/6J mouse suffering from EAU. Gating strategy for M1/M2 macrophages using cells isolated from ileum of healthy placebo-treated C57BL/6J mouse (D).

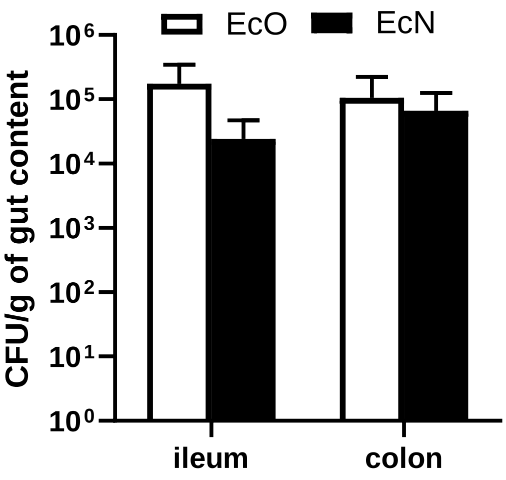

Figure 2. Probiotic EcO and EcN colonize mice used in our experiments for at least 48 h after a single dose. The results were calculated by comparing threshold cycle with a standard curve constructed from known number of CFU for each bacterium and then related to the sample weight.

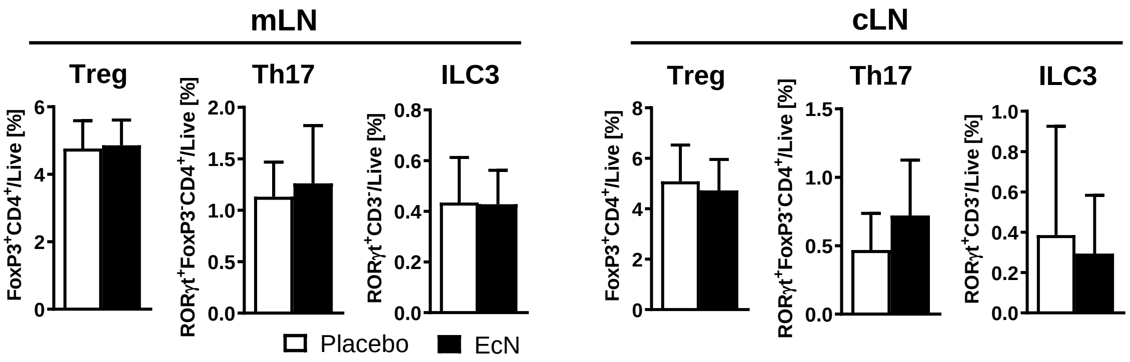

Figure 3. Live probiotic EcN does not change proportion of regulatory T cells, Th17 cells or innate lymphoid cells (ILC) 3 in mLN or cLN at day 28 post-induction. (n = 22 (placebo), 23 (EcN) in total).

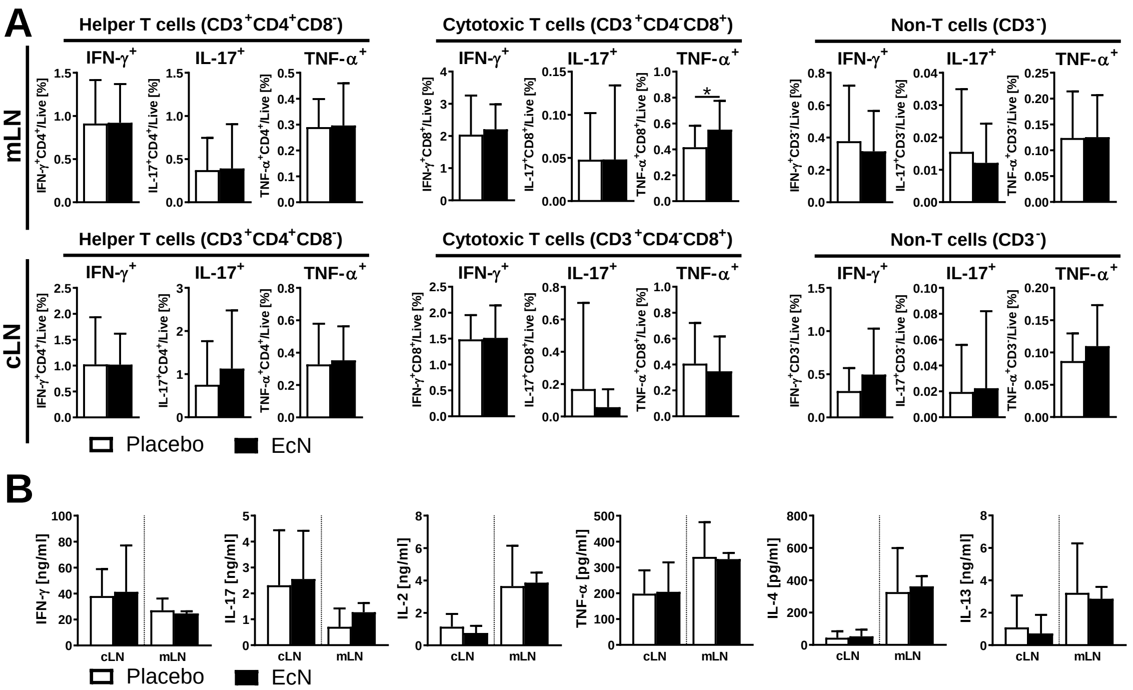

Figure 4. Oral EcN has no effect on expression of pro-inflammatory intracellular cytokine of anti-CD3/CD28 activated helper or cytotoxic T cells in mLN or cLN, as measured by FACS (A). In addition, there is no accumulation of T cell cytokines in supernatants of activated T cells isolated from mLN and cLN, as measured by ELISA (B). Differences were quantified by unpaired Mann-Whitney test; *$p<0.05$. Data are pools from 5 independent experiments ($n = 24$ per group in total). Data are from day 28 post-induction.

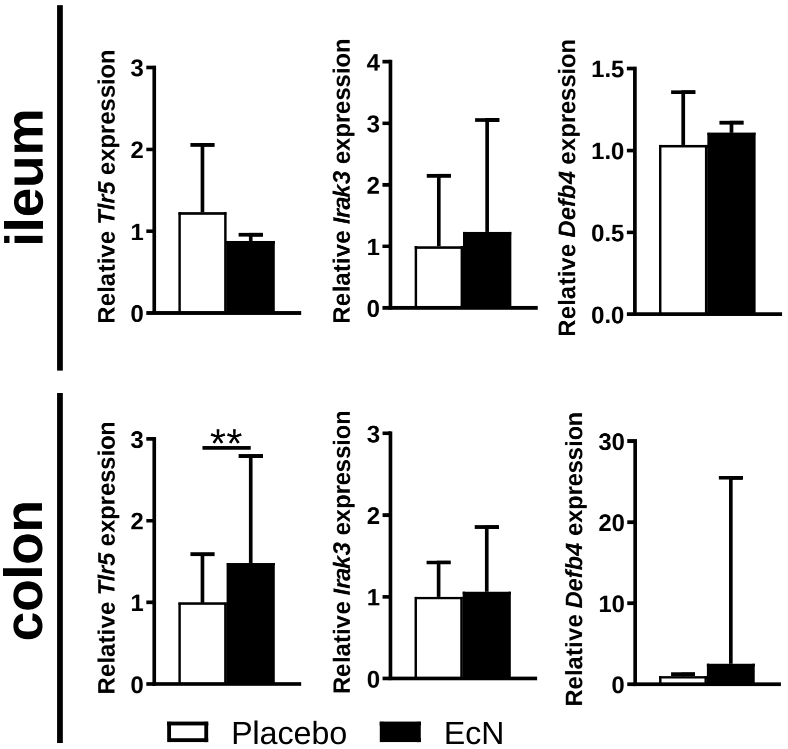

Figure 5. Live EcN increases the expression of tlr5 in the colon at day 28 post-induction. Data are pool from 5 independent experiments (n = 3-8/group) and differences were quantified by unpaired Mann-Whitney test; *p<0.05 **p<0.01.

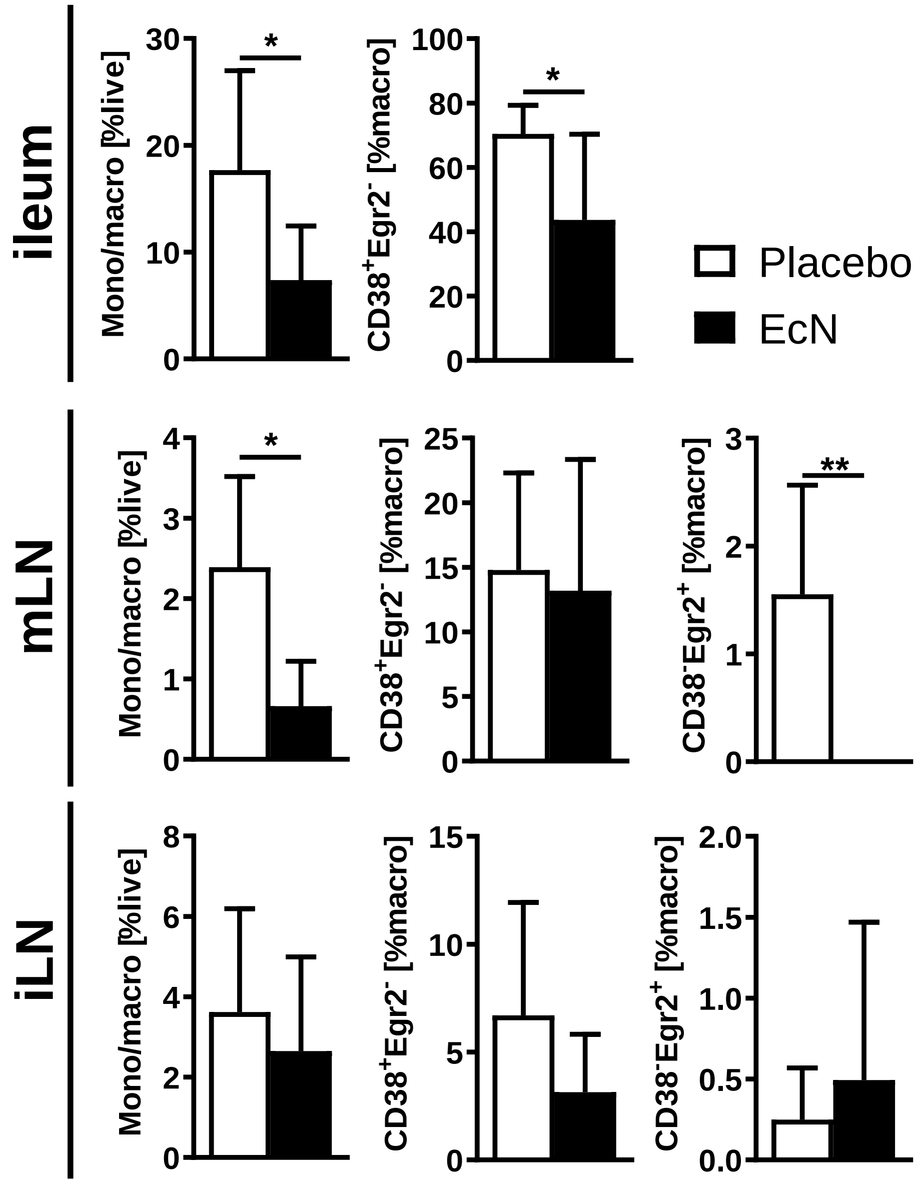

Figure 6. Live EcN decreases proportion of macrophages and shift their M1 at day 7 post-induction. This effect is clearly apparent in the ileum (A). The trend is still visible in mesenteric lymph nodes (B) and in inguinal lymph nodes (C). Differences were quantified by unpaired Mann-Whitney test (n = 9 (placebo) or 7 (EcN)); *p<0.05, **p<0.01. The macrophages were defined as live $CD45^+F4/80^+CD11b^+$ cells and M1 ($CD38^+Egr2^+$) and M2 ($CD38^-Egr2^+$) markers were selected according to previously published data [1] Ileum did not contained clear M2 population.

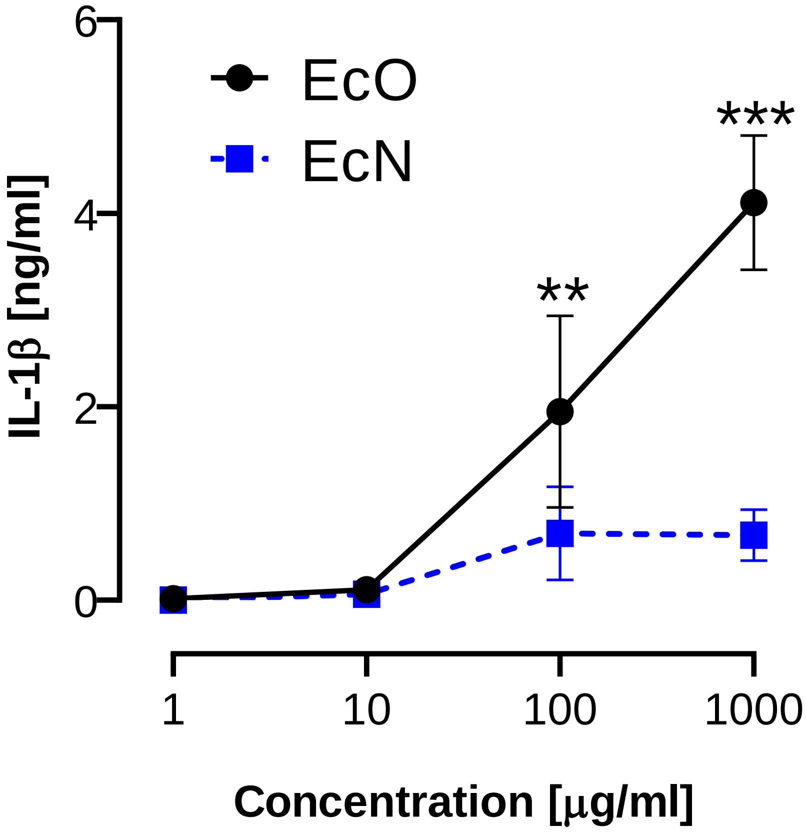

Figure 7. EcO lysate induces higher IL-1β production in BMDM compared to EcN lysate. The differences between EcO (n = 4) and EcN (n = 4) were analyzed by 2-way ANOVA with Bonferroni's multiple comparisons test (n = 4); **p<0.01, ***p<0.001

Table 1. List of antibodies.

**Epitope – Fluorochrome**	**Clone**	**Manufacturer**	**Cat#**
CD3ε (purified)	145-2C11	BioLegend	100359
CD28 (purified)	37.51	BioLegend	102121
CD16/32 (purified)	93	BioLegend	101302
Fixable Viability Dye eFluor 780[1, 2, 3, 4]	-	Thermo Fisher Scientific	65-0865-14
CD3 – eFluor 450[3]	17A2	Thermo Fisher Scientific	48-0032-82
CD3ε – FITC[1, 2]	145-2C11	BioLegend	100306
CD4 – Brilliant violet 605[1, 2]	GK1.5	BioLegend	100451
CD8 – Brilliant violet 650[1, 2]	53-6.7	BioLegend	100741
CD11b – Brilliant violet 605[4]	M1/70	BioLegend	1106285
CD11c – Brilliant violet 711[3]	N418	BioLegend	117349
CD25 – APC[1]	PC61.5	Thermo Fisher Scientific	17-0251-82
CD38 – FITC[4]	90	BioLegend	102705
CD40 – PE[3]	1C10	Thermo Fisher Scientific	12-0401-82
CD45 – Alexa Fluor 700[3, 4]	30-F11	BioLegend	103128
B220 (CD45R) – Brilliant violet 510[3]	RA3-6B2	BioLegend	103247
CD49b – eFluor 450[3]	DX5	Thermo Fisher Scientific	48-5971-82
Egr2 – APC[4]	erongr2	Thermo Fisher Scientific	17-6691-82
F4/80 – PE[4]	BM8	BioLegend	123110
Foxp3 – PE[1]	FJK-16s	Thermo Fisher Scientific	12-5773-82
IFN-γ – PE[1, 2]	XMG1.2	Thermo Fisher Scientific	12-7311-81
IL-17A – APC[1, 2]	eBio17B7	Thermo Fisher Scientific	17-7177-81
iNOS – PE-Cyanine7[3]	CXNFT	Thermo Fisher Scientific	25-5920-80
Ly-6C – Brilliant violet 605[3]	HK1.4	BioLegend	128036
Ly-6G – FITC[3]	1A8	BioLegend	127605
Rorγt – Brilliant violet 421[1]	Q31-378	BD Biosciences	562894
TNF-α– PE-Cyanine7[1, 2]	TN3-19.12	Thermo Fisher Scientific	25-7423-82

Used to analyze: [1] regulatory T cells, Th17 and ILC3, [2] cytokine production by T cells, [3] monocytes and macrophages or [4] M1/M2 monocytes and macrophages.

Table 2. Primers used in qPCR and RT-PCR.

Target	Forward primer (5′–>3′)	Reverse primer (5′–>3′)	Reference
EcN	AACTGTGAAGCGATGAACCC	GGACTGTTCAGAGAGCTATC	[2]
EcO	CTAATGCGGGCAGAGAAATAAAGT	ATAAAGACGGCAGGGTAACACAC	[3]
Defb4	ATCTCACCAGGCTTCAGT	TCTTCATGGAGGAAATTC	this study
Tlr5	CAGGATGTTGGCTGGTTTCT	CGGATAAAGCGTGGATT	[4]
Irak3	TTGGTCCTGGGCACAGAAA	AATAGCTCGACGATGTCCCAT	[5]
pan-Defensin α	GGTGATCATCAGACCCCAGCATCAGT	AAGAGACTAAAACTGAGGAGCAGC	[6]
Defa5	TCCTGCTCAACAATTCTCCA	GACACAGCCTGGTCCTCTTC	[7]
S100a8	AAATCACCATGCCCTCTACAAG	CCCACTTTTATCCCATCGCAAA	[8]
S100a9	GGCCAACAAAGCACCTTCTCA	CTTGCTCAGGGTGTCAGGG	this study
Reg3β	CTGCCTTAGACCGTGCTTTC	CCCTTGTCCATGATGCTCTT	[9]
Reg3γ	TTCCTGTCCTCCATGATCAAAA	CATCCACCTCTGTTGGGTTCA	[10]
Muc13	GAGGAGAAACAGGAGCATAG	GGACATCAAGGGAGAAG	this study
Tjp1 (ZO-1)	CCACCTCTGTCCAGCTCTTC	CACCGGAGTGATGGTTTTCT	[11]
Eef2	CTGACACTCGCAAGGATGAG	CCCGGTCCATCTTGTTCATC	[12]

Supplementary References:

1. Jablonski, K.A.; Amici, S.A.; Webb, L.M.; Ruiz-Rosado Jde, D.; Popovich, P.G.; Partida-Sanchez, S.; Guerau-de-Arellano, M. Novel Markers to Delineate Murine M1 and M2 Macrophages. *PLoS ONE* **2015**, *10*, e0145342, doi:10.1371/journal.pone.0145342.
2. Blum-Oehler, G.; Oswald, S.; Eiteljorge, K.; Sonnenborn, U.; Schulze, J.; Kruis, W.; Hacker, J. Development of strain-specific PCR reactions for the detection of the probiotic Escherichia coli strain Nissle 1917 in fecal samples. *Res. Microbiol.* **2003**, *154*, 59–66, doi:10.1016/s0923-2508(02)00007-4.
3. Hejnova, J.; Dobrindt, U.; Nemcova, R.; Rusniok, C.; Bomba, A.; Frangeul, L.; Hacker, J.; Glaser, P.; Sebo, P.; Buchrieser, C. Characterization of the flexible genome complement of the commensal Escherichia coli strain A0 34/86 (O83 : K24 : H31). *Microbiology* **2005**, *151*, 385–398, doi:10.1099/mic.0.27469-0.
4. Monteleone, I.; Platt, A.M.; Jaensson, E.; Agace, W.W.; Mowat, A.M. IL-10-dependent partial refractoriness to Toll-like receptor stimulation modulates gut mucosal dendritic cell function. *Eur. J. Immunol.* **2008**, *38*, 1533–1547, doi:10.1002/eji.200737909.
5. Standiford, T.J.; Kuick, R.; Bhan, U.; Chen, J.; Newstead, M.; Keshamouni, V.G. TGF-beta-induced IRAK-M expression in tumor-associated macrophages regulates lung tumor growth. *Oncogene* **2011**, *30*, 2475–2484, doi:10.1038/onc.2010.619.
6. Gulati, A.S.; Shanahan, M.T.; Arthur, J.C.; Grossniklaus, E.; von Furstenberg, R.J.; Kreuk, L.; Henning, S.J.; Jobin, C.; Sartor, R.B. Mouse background strain profoundly influences Paneth cell function and intestinal microbial composition. *PLoS ONE* **2012**, *7*, e32403, doi:10.1371/journal.pone.0032403.
7. Buczacki, S.J.; Zecchini, H.I.; Nicholson, A.M.; Russell, R.; Vermeulen, L.; Kemp, R.; Winton, D.J. Intestinal label-retaining cells are secretory precursors expressing Lgr5. *Nature* **2013**, *495*, 65–69, doi:10.1038/nature11965.
8. Zhou, J.; Wu, R.; High, A.A.; Slaughter, C.A.; Finkelstein, D.; Rehg, J.E.; Redecke, V.; Hacker, H. A20-binding inhibitor of NF-kappaB (ABIN1) controls Toll-like receptor-mediated CCAAT/enhancer-binding protein beta activation and protects from inflammatory disease. *Proc. Natl. Acad. Sci. USA* **2011**, *108*, E998-1006, doi:10.1073/pnas.1106232108.
9. Jacobson, A.; Lam, L.; Rajendram, M.; Tamburini, F.; Honeycutt, J.; Pham, T.; Van Treuren, W.; Pruss, K.; Stabler, S.R.; Lugo, K.; et al. A Gut Commensal-Produced Metabolite Mediates Colonization Resistance to Salmonella Infection. *Cell Host Microbe* **2018**, *24*, 296–307 e297, doi:10.1016/j.chom.2018.07.002.
10. Syder, A.J.; Oh, J.D.; Guruge, J.L.; O'Donnell, D.; Karlsson, M.; Mills, J.C.; Bjorkholm, B.M.; Gordon, J.I. The impact of parietal cells on Helicobacter pylori tropism and host pathology: An analysis using gnotobiotic normal and transgenic mice. *Proc. Natl. Acad. Sci. USA* **2003**, *100*, 3467–3472, doi:10.1073/pnas.0230380100.
11. Volynets, V.; Rings, A.; Bardos, G.; Ostaff, M.J.; Wehkamp, J.; Bischoff, S.C. Intestinal barrier analysis by assessment of mucins, tight junctions, and alpha-defensins in healthy C57BL/6J and BALB/cJ mice. *Tissue Barriers* **2016**, *4*, e1208468, doi:10.1080/21688370.2016.1208468.
12. Stehlikova, Z.; Kostovcikova, K.; Kverka, M.; Rossmann, P.; Dvorak, J.; Novosadova, I.; Kostovcik, M.; Coufal, S.; Srutkova, D.; Prochazkova, P.; et al. Crucial Role of Microbiota in Experimental Psoriasis Revealed by a Gnotobiotic Mouse Model. *Front. Microbiol* **2019**, *10*, 236, doi:10.3389/fmicb.2019.00236.
